# Salience Network Segregation Mediates the Effect of Tau Pathology on Mild Behavioral Impairment

**DOI:** 10.1002/alz.094737

**Published:** 2025-01-09

**Authors:** Alexandru D Iordan, Robert Ploutz‐Snyder, Bidisha Ghosh, Annalise Rahman‐Filipiak, Robert A. Koeppe, Scott J Peltier, Bruno Giordani, Roger L. Albin, Benjamin M. Hampstead

**Affiliations:** ^1^ University of Michigan, Ann Arbor, MI USA

## Abstract

**Background:**

The recently developed construct of mild behavioral impairment (MBI) provides a diagnostic framework for early signs of behavioral dysfunction that may appear even before other symptoms of cognitive decline and dementia in older adults. However, the links between MBI, brain function, and Alzheimer’s disease (AD) biomarkers are unclear.

**Method:**

Using data from 128 participants with diagnosis of amnestic mild cognitive impairment or mild dementia of the Alzheimer’s type, we test a novel model assessing direct relationships between AD biomarker status (amyloid and tau PET) and MBI symptoms, as well as mediated effects through the functional segregation of the salience, default‐mode, and frontoparietal control networks from the other high‐level, association networks.

**Result:**

We identified a mediated effect of tau positivity on MBI through functional segregation of the salience network from the other association networks. Specifically, individuals who were positive for both amyloid and tau showed lower overall segregation of the salience network compared to those who were AD biomarker negative (β = ‐0.25, *p* = 0.007). Lower overall segregation of the salience network was associated with more severe MBI symptoms (β = ‐0.441, *p* = 0.013). There were no direct effects of AD biomarkers status on MBI.

**Conclusion:**

Our findings suggest that the effect of tau pathology on MBI may operate through the modulation of salience network functional integrity. This mediated relationship was unique for the salience network since including the default‐mode and frontoparietal control networks in our models did not change the tau‐specific findings. To the best of our knowledge, this is the first study to integrate MBI with AD PET biomarkers and network‐level brain functionality within a unified framework.

